# Factors associated with postoperative shivering in patients with maintained core temperature after surgery

**DOI:** 10.1186/s40981-024-00755-8

**Published:** 2024-11-11

**Authors:** Kazuhiro Shirozu, Masako Asada, Ryotaro Shiraki, Takuma Hashimoto, Ken Yamaura

**Affiliations:** 1 Operating Rooms, Kyushu University Hospital, 3-1-1 Maidashi, Higashi-ku, Fukuoka, 812-8582 Japan; 2 Department of Anesthesiology and Critical Care Medicine, Kyushu University Graduate School of Medicine, Fukuoka, Japan; 3 Department of Anesthesiology and Critical Care Medicine, Kyushu University Hospital, Fukuoka, Japan

**Keywords:** Shivering, Peripheral temperature, Core temperature, Acetaminophen

## Abstract

**Background:**

Postoperative shivering is mainly associated with low body temperature. However, postoperative shivering can develop even at normal or high core temperatures. This study aimed to investigate the factors associated with postoperative shivering in patients with maintained core temperature after surgery.

**Methods:**

This retrospective study involved 537 patients who had undergone radical surgery for pancreatic cancer under general anesthesia from January 2013 to December 2023. The final analysis included 441 patients whose core temperatures after surgery were ≥ 36.5℃. Logistic regression analysis was performed to estimate the odds ratio (OR) of the incidence of postoperative shivering.

**Results:**

Postoperative shivering occurred in 119 patients. After multivariable-adjusted logistic regression, postoperative shivering was significantly associated with patient age (per 1 year increase; OR = 0.98; 95% confidence interval [CI]: 0.96–0.996; *p* = 0.02), operation time (per 30 min increase; OR = 1.10; 95% CI: 1.01–1.19; *p* = 0.03), postoperative core temperature (restricted cubic spline, *p* = 0.001), postoperative peripheral temperature (restricted cubic spline, *p* = 0.001), effect site fentanyl concentration at extubation (OR = 0.66; 95% CI: 0.24–0.99; *p* = 0.049), and acetaminophen use (OR = 0.32; 95% CI: 0.18–0.58; *p* < 0.001).

**Conclusions:**

Low peripheral temperature was a risk factor for the occurrence of shivering, even if the core temperature was maintained postoperatively. Peripheral temperature monitoring could be utilized to prevent postoperative shivering. In addition, fentanyl and acetaminophen reduced the occurrence of shivering in patients with maintained core temperature after surgery.

## Introduction

Postoperative shivering should be prevented as it increases oxygen consumption and catecholamine levels [1]. To prevent postoperative shivering, drugs that reduce thermoregulated vasoconstriction and shivering thresholds [2] and warming devices can be used [3, 4]. Magnesium, ondansetron, and acetaminophen administration during anesthesia are used to prevent postoperative shivering [5–8]. According to the clinical guidelines of the National Institute for Health and Care Excellence, all patients requiring surgery should be warmed with a forced-air warming system or warming blanket from the induction of anesthesia to the end of surgery. The guideline also recommended that patients’ core temperature should be maintained at ≥ 36.5℃ and ≤ 37.5℃ [9]. However, postoperative shivering is detected even in patients whose core temperature is maintained according to above criteria after surgery. On the other hand, peripheral temperature is also known to be related to postoperative shivering but is not used or utilized for postoperative shivering prevention in many facilities. To the best of our knowledge, no reports have examined the factors affecting the incidence of postoperative shivering in patients with maintained postoperative core temperature. Therefore, the primary purpose of this study was to explore factors associated with postoperative shivering in patients with a core temperature ≥ 36.5℃ after surgery. We chose cases of radical open pancreatic surgery because it makes the threshold for shivering relatively high owing to the large surgical invasion and is associated with a relatively high incidence of postoperative shivering.

## Materials

### Patients and study design

This observational, nonrandomized, retrospective study was approved by the Institutional Clinical Research Ethics Committee of Kyushu University, Fukuoka, Japan (IRB: Clinical Research number #23346–00) and individual written informed consent was waived by the above-mentioned institutional review board. All study protocols were compliant with the Declaration of Helsinki (2013). We enrolled 537 patients who were scheduled to undergo radical surgery for pancreatic cancer at our university hospital between January 2013 and December 2023. Patients with endocrine diseases affecting body temperature, laparoscopic surgery cases, and patients with data defects and under 20 years old were excluded. Data were extracted using a data warehouse (DWH) mining tool (Nihon Kohden, Tokyo, Japan). Data on patient age, body mass index (BMI), operation time, anesthesia time, anesthesia technique (total intravenous anesthesia or volatile anesthesia), administration of flurbiprofen/acetaminophen/pethidine during surgery, amount of bleeding or urine, fentanyl/remifentanil effect site concentration, core temperature, and peripheral (fingertip) temperature were obtained. A shivering incidence was defined as a case of pethidine administration after extubation or a recorded case of shivering as a complication in the electronic anesthesia chart. We regarded muscle activity in more than one muscle as shivering and administrated pethidine for shivering. After insertion of the epidural catheter, anesthesia was induced by the intravenous administration of fentanyl, propofol, and rocuronium, and it was maintained with desflurane (4–5%), sevoflurane (1.5–2%) or propofol (target-controlled infusion of 3–4 µg/mL), remifentanil, and a 40–50% oxygen–air mixture. Additional bolus infusions of fentanyl and rocuronium were administered, as required. To manage postoperative pain, anesthesiologists administered acetaminophen, flurbiprofen, or fentanyl, except for a bolus infusion of epidural anesthesia, before the end of surgery. No opioids were given into epidural anesthesia intra and post-operative period. At the discretion of the anesthesiologist in charge, pethidine was used intra-operatively in a few cases postoperative shivering was expected to occur. Anesthesiologists focused on a target mean blood pressure of 60–70 mmHg during the surgery. For body warming, the double lumen fluid warming system (HOTLINE®, smith medical, Plymouth, MN, USA) and the forced-air warming device (Bair Hugger™ 775 & blanket 522, 3 M, St. Paul, MN, USA) were used for all patients. Warming blanket was covered the upper body at 38–43 ℃ setting, and ambient temperature was set at 26℃ with 40% humidity during surgery and at 28℃ with 50% humidity after surgery.

### Statistical analysis

Data are presented as mean (standard deviation) or median (interquartile range) for continuous variables or as percentages for categorical variables. We compared the baseline characteristics between the shivering and non-shivering groups using unpaired t-test, chi-square test, Mann–Whitney U test, or Fisher exact test, as appropriate. Multivariable-logistic regression analysis was performed to estimate the odds ratios (ORs) with 95% confidence intervals (CIs) of postoperative shivering. Variables that did not show a linear relationship with the logit of the outcome were transformed as restricted cubic splines. In the logistic regression, restricted cubic spline with 3 knots located at the 10th, 50th, and 90th percentiles of the variable distribution were used. In the multivariate-adjusted analysis, patient age [7], sex, BMI, operation time, amount of bleeding, postoperative (just after surgery) core and peripheral temperature [10], fentanyl effect site concentration [11], epidural anesthesia [12], anesthesia technique (total intravenous or volatile anesthesia) [13], and administration of flurbiprofen/acetaminophen/pethidine [7, 14, 15] were included in the model to control for confounding factors. These candidates factors have been reported in previous study to be associated with postoperative shivering. The independence of these variables was checked for multicollinearity. Moreover, spline curves were used to visualize the relationship of peripheral temperature, core-peripheral temperature difference, and core temperature with the risk of postoperative shivering. SAS software package (version 9.4; SAS Institute, Cary, NC, USA) was used for all statistical analyses. Two-sided values of *p* < 0.05 were considered statistically significant in all analyses.

## Results

### Baseline characteristics of the participants

**Fig. 1:**
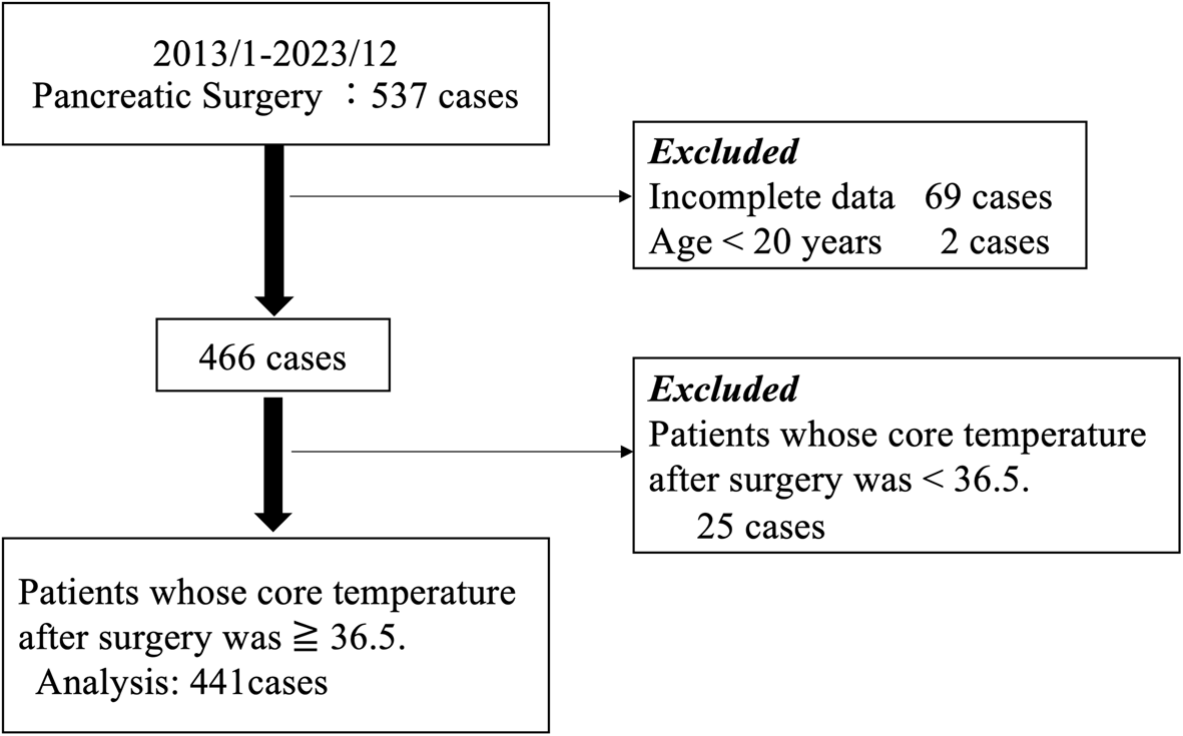
Flow diagram

Among 537 patients, 69 patients whose data was incomplete and 25 patients whose core temperature after surgery was < 36.5℃, and 2 patients whose age was under 20 were excluded (Fig. 1). Finally, 441 patients were analysed, among whom 119 developed postoperative shivering. Patients in the shivering group were significantly younger than those in the non-shivering group (64.8 ± 13.7 vs. 67.5 ± 10.7 years, *p* = 0.03). The proportion of men were significantly higher in the shivering group than in the non-shivering group (69.8 vs. 58.7%, *p* = 0.03). The duration of anesthesia was longer in the shivering group than in the non-shivering group (498 [420–592] vs. 456 [393–523] min, *p* < 0.001). Similarly, the operation time was longer in the shivering group than in the non-shivering group (425 min [352–518 min] vs. 387 min [323–448 min], *p* < 0.001). The median amount of bleeding was significantly higher in the shivering group than in the non-shivering group (810 [500–1090] vs. 611 [394–917] mL, *p* = 0.002), the median urinary output was significantly higher in the shivering group than in the non-shivering group (500 [300–800] vs. 400 [200–700] mL, *p* = 0.04), and the median volume of infusion was significantly greater in the shivering group than in the non-shivering group (4345 [3439–5167] vs. 3839 [2952–4652] mL, *p* < 0.001). The postoperative peripheral temperature was much lower in the shivering group than in the non-shivering group (34.4 [32.1–35.8] vs. 35.4 [33.0–36.4] ℃, *p* = 0.001). The difference between the postoperative core and peripheral temperatures was significantly greater in the shivering group than in the non-shivering group (3.3 [1.7–5.3] vs. 2.4 [1.3–4.7] ℃, *p* = 0.002). The median effect site fentanyl concentration at extubation was significantly higher in the shivering group than in the non-shivering group (0.80 ± 0.33 vs. 0.90 ± 0.37 ng/mL, *p* = 0.01). The proportion of flurbiprofen users was much more in the shivering group than in the non-shivering group (63.0 vs. 48.8%; *p* = 0.008). The proportion of acetaminophen users was much lesser in the shivering group than in the non-shivering group (44.5 vs. 66.8%; *p* < 0.001). The proportion of pethidine users intraoperatively was much more in the shivering group than in the non-shivering group (16.8 vs. 3.1%; *p* < 0.001). Other factors were nearly identical between the groups (Table 1).

**Table 1:** Comparison of baseline characteristics between the shivering and non-shivering groups

	Shivering ( +)	Shivering (-)	*p* value*
	(*n* = 119)	(*n* = 322)	
Age, years	64.8 (13.7)	67.5 (10.7)	0.03
Men, %	69.8	58.7	0.03
Body mass index, kg/m^2^	22.2 (3.4)	21.7 (3.0)	0.12
Anesthesia time, min	498 (420–592)	456 (393–523)	< 0.001^a^
Operation time, min	425 (352–518)	387 (323–448)	< 0.001^a^
Amount of bleeding, mL	810 (500–1090)	611 (394–917)	0.002^a^
Urinary output, mL	500 (300–800)	400 (200–700)	0.04
Volume of infusion, mL	4345 (3439–5167)	3839 (2952–4652)	< 0.001^a^
Postoperative core temperature, °C	37.6 (37.1–38.1)	37.6 (37.3–38.0)	0.94^a^
Difference between postoperative core and peripheral temperature, °C	3.3 (1.7–5.3)	2.4 (1.3–4.7)	0.002^a^
Postoperative peripheral temperature, °C	34.4 (32.1–35.8)	35.4 (33.0–36.4)	0.001^a^
Fentanyl effect site concentration, ng/mL	0.80 (0.33)	0.90 (0.37)	0.01
Remifentanil effect site concentration, ng/mL	0.67 (0.74)	0.62 (0.68)	0.54
Epidural anesthesia, %	95.4	91.0	0.10
Total intravenous anesthesia, %	4.2	2.2	0.18^b^
Use of flurbiprofen, %	63.0	48.8	0.008
Use of acetaminophen, %	44.5	66.8	< 0.001
Use of pethidine during surgery, %	16.8	3.1	< 0.001

Values are presented as means (standard deviation [SD]), medians (interquartile range [IQR]), or percentages

^*^p values are based on the unpaired t-test or chi-square test, unless otherwise specified

^a^Mann–Whitney U test

^b^Fisher's exact test

### Associated factors of postoperative shivering in a multivariable-adjusted analysis

In the crude model, the ORs for the incidence of postoperative shivering were significantly associated with patient age (per 1 year increase; OR = 0.98; 95% CI: 0.96–0.998; *p* = 0.03), male patients (OR = 1.62; 95% CI: 1.04–2.54; *p* = 0.04), operation time (per 30 min increase; OR = 1.12; 95% CI: 1.05–1.18; *p* < 0.001) and amount of bleeding (per 100 mL increase; OR = 1.04; 95% CI: 1.005–1.07), postoperative core temperature (restricted cubic spline, *p* = 0.02), postoperative peripheral temperature (restricted cubic spline, *p* = 0.02), fentanyl effect site concentration (OR = 0.43, 95% CI: 0.23–0.82, *p* = 0.01), use of flurbiprofen (OR = 1.80, 95% CI: 1.16–2.76, *p* = 0.008), and use of acetaminophen (OR = 0.40, 95% CI: 0.26–0.61, *p* < 0.001). Other factors were not associated with postoperative shivering. These associations were almost the same in the multivariable-adjusted model, except for sex, amount of bleeding, epidural anesthesia, and use of flurbiprofen. Patient age (OR = 0.98; 95% CI: 0.96–0.996; *p* = 0.02), operation time (OR = 1.10; 95% CI: 1.01–1.19; *p* = 0.03), postoperative core temperature (restricted cubic spline, *p* = 0.001), postoperative peripheral temperature (restricted cubic spline, *p* = 0.001), epidural anesthesia (OR = 3.39, 95% CI: 1.12–10.23, *p* = 0.03), fentanyl effect site concentration (OR = 0.66, 95% CI: 0.24–0.99, *p* = 0.048), and administration of acetaminophen (OR = 0.32; 95% CI: 0.18–0.58; *p* < 0.001) were significantly associated with postoperative shivering (Table 2). In the multivariable-adjusted model, c-statistics was 0.750.

**Table 2:** Crude and multivariable-adjusted odds ratio for postoperative shivering

Variables	Crude		Multivariable-adjusted^a^	
	OR (95% CI)	*p* value	OR (95% CI)	*p* value
Age, year	0.98 (0.96–0.998)	0.03	0.98 (0.96–0.996)	0.02
Men	1.62 (1.04–2.54)	0.04	1.53 (0.91–2.55)	0.11
Body mass index, kg/m^2^	1.05 (0.99–1.13)	0.12	1.00 (0.92–1.08)	0.93
Operation time, per 30 min	1.12 (1.05–1.18)	< 0.001	1.10 (1.01–1.19)	0.03
Amount of bleeding, per 100 mL	1.04 (1.005–1.07)	0.03	1.03 (0.99–1.08)	0.317
Postoperative core temperature, °C	Not available	0.02	Not available	0.001
Postoperative peripheral temperature, °C	Not available	0.02	Not available	0.001
Fentanyl effect site concentration, ng/mL	0.43 (0.23–0.82)	0.01	0.66 (0.24–0.99)	0.049
Epidural anesthesia				
−	Reference		Reference	
+	2.26 (0.85–5.97)	0.13	3.39 (1.12–10.23)	0.03
Anesthesia				
Volatile	Reference		Reference	
Total intravenous anesthesia	2.31 (0.69–7.72)	0.17	2.24 (0.62–8.05)	0.22
Flurbiprofen				
−	Reference		Reference	
+	1.80 (1.16–2.76)	0.008	1.19 (0.68–2.08)	0.53
Acetaminophen				
−	Reference		Reference	
+	0.40 (0.26–0.61)	< 0.001	0.32 (0.18–0.58)	< 0.001

*OR* indicates odds ratio, *CI* confidence interval

^a^c-statistic 0.747

### Relationship between peripheral temperature, difference between core and peripheral temperatures, and core temperature with postoperative shivering

**Fig. 2:**
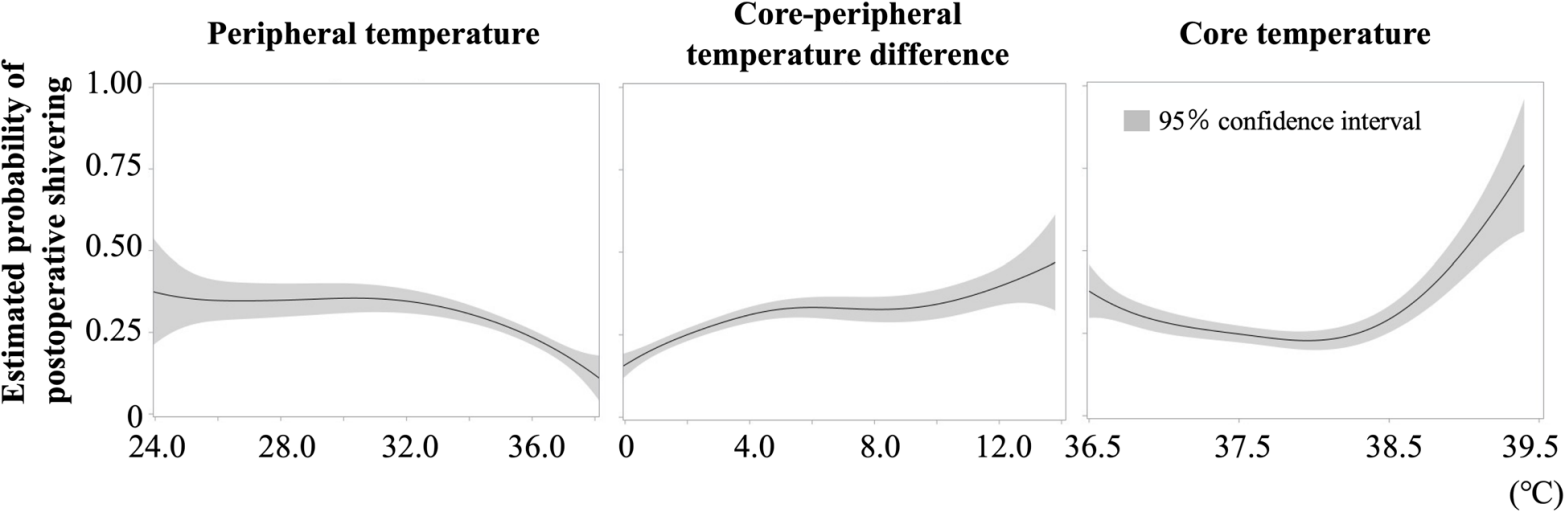
Relationships of peripheral temperature (left), core-peripheral temperature difference (centre), and core temperature (right) with postoperative shivering. The predicted value of postoperative shivering was calculated using a logistic regression model. The gray ranges indicate 95% confidence intervals

Figure 2 demonstrates the relationship between postoperative temperature and postoperative shivering. Estimated probability of postoperative shivering decreased as postoperative peripheral temperature increased above 32.0℃ (Fig. 2, left). Contrastingly, the predicted value of postoperative shivering decreased as core-peripheral temperature difference decreased below 4.0℃ (Fig. 2, center). Regarding the postoperative core temperature above 38.0℃, the predicted value of postoperative shivering increased (Fig. 2, right).

## Discussion

Core temperature during surgery can be maintained to prevent postoperative shivering. However, this study showed that attention should be paid to maintaining the peripheral temperature while maintaining the core body temperature. Specifically, the peripheral temperature should be kept above 32.0 °C. Although patient age and operative time are irrelevant to anaesthesiologist management, peripheral temperature management, and intraoperative fentanyl and acetaminophen use to prevent postoperative shivering is depended on the anesthesiologist.

The increased difference between peripheral and core temperatures can also be caused by the vasoconstriction that occurs during thermoregulation, which is also a precursor for shivering. Thermoregulatory vasoconstriction is suspected at above3.0–4.0 °C in skin-surface temperature gradients (forearm temperature-fingertip temperature) [16]. In this study, the risk of shivering also decreased linearly when core-peripheral temperature difference was less than 4.0 °C. Additionally, peripheral temperatures above 32.0 °C decreased the risk of shivering occurrence. These results suggested that the risk of shivering decreases when the peripheral temperature is as much as possible above 32.0 °C in core temperature increasing cases. Peripheral temperature is associated with the incidence of shivering [10, 17]. Postoperative shivering and vasoconstriction are contributed by about 20% skin and 80% core temperatures [10]. Therefore, peripheral temperature monitoring is clinically important, and the extremities should be kept as warm as the trunk. Peripheral skin temperature is affected by the sympathetic nerve status and fluid balance, as well as external temperature. Then, pain and circulation management are also related to postoperative shivering prevention. Alternatively, the significant increase in the risk of shivering in a core temperature above 38.0 °C may be due to a vasoconstriction reaction for thermoregulation. The core temperature may have risen too high owing to continuous warming caused by a low peripheral temperature. At this point, continued warming of the trunk does not reduce the risk of shivering. Therefore, once the core temperature reaches ≥ 38.0 °C, we should recognize the possibility that peripheral circulation is affected by vasoconstriction or insufficient heating of the peripheral sites, and the risk of shivering might be high.

Acetaminophen lowers the threshold for shivering [18], exerts a vasodilating effect [19], and increases skin blood flow [20]. However, peripheral temperature after surgery was significantly lower in patients who were administered acetaminophen (*n* = 268) than in those who were not administered acetaminophen (*n* = 173; 34.7 [31.8–35.9] vs. 35.7 [34.2–36.5] ℃, *p* < 0.001) (not shown by table or figure). This result indicates that the preventive effect of acetaminophen on shivering may be mainly due to the threshold for the shivering-lowering effect in this study. These results indicate that acetaminophen is useful in preventing postoperative shivering at low peripheral temperatures.

Additionally, this study revealed that increasing effect site concentration of fentanyl at extubation could be effective in reducing shivering. This result was reasonable becase opioids and sulfentanil have been reported to inhibit shivering [11, 14]. However, fentanyl should be administered with caution in terms of delayed arousal, then, a higher dose is not always better in clinical setting.

On the other hand, there is room for consideration in the result that the proportion of epidural anesthesia was much more in the shivering group than in the non-shivering group. Because confidence interval was wide (1.12–10.23) and spinal block height has been reported to decrease the shivering threshold [12]. This result may be attributed to the fact that many cases were managed with epidural anesthesia (407 vs. 34 cases without epidural anesthesia), or reflect the play of chance.

This study has certain limitations that must be addressed. The main study limitation is its retrospective design. Although shivering cases were defined as cases for which shivering was recorded as an intraoperative complication in the electronic anesthesia chart, misclassification may have occurred. Moreover, we only examined the postoperative shivering during the approximately 30 min between extubation and discharge, it could have occurred in the ward. This may have weakened the association observed in this study. Additionally, pethidine was mostly administered for treatment but sometimes for the prevention of shivering in our institute. Then, we regarded the case administed pethidine after extubation as postoperative shivering development case. The petidine administered intraoperatively was presumably given as prophylaxis in anticipation of shivering, and was shown in Table 1.This was reason why the proportion of pethidine users was much more in the shivering group than in the non-shivering group (Table 1). Finally, this study targeted only open pancreatic surgery, which caused a significant shift in shivering thresholds. Therefore, the results of the present study may not apply to all surgical procedures.

## Conclusions

This study indicated that raising the core temperature alone did not reduce the incidence of postoperative shivering. Peripheral temperature monitoring could be effective in preventing postoperative shivering. To prevent shivering, it is necessary not to only keep the body warm but also control pain and body fluid balance to prevent peripheral blood vessels from excessive constriction. Furthermore, the use of acetaminophen can help prevent postoperative shivering, even in patients with a high core temperature.

## Data Availability

Not applicable.
